# Osteoporosis Associated with Antipsychotic Treatment in Schizophrenia

**DOI:** 10.1155/2013/167138

**Published:** 2013-04-17

**Authors:** Haishan Wu, Lu Deng, Lipin Zhao, Jingping Zhao, Lehua Li, Jindong Chen

**Affiliations:** ^1^Institute of Mental Health, Second Xiangya Hospital, Central South University, Changsha 410011, China; ^2^Department of Nursing, Second Xiangya Hospital, Central South University, Changsha 410011, China

## Abstract

Schizophrenia is one of the most common global mental diseases, with prevalence of 1%. Patients with schizophrenia are predisposed to diabetes, coronary heart disease, hypertension, and osteoporosis, than the normal. In comparison with the metabolic syndrome, for instance, there are little reports about osteoporosis which occurs secondary to antipsychotic-induced hyperprolactinaemia. There are extensive recent works of literature indicating that osteoporosis is associated with schizophrenia particularly in patients under psychotropic medication therapy. As osteoporotic fractures cause significantly increased morbidity and mortality, it is quite necessary to raise the awareness and understanding of the impact of antipsychotic-induced hyperprolactinaemia on physical health in schizophrenia. In this paper, we will review the relationship between schizophrenia, antipsychotic medication, hyperprolactinaemia, and osteoporosis.

## 1. Introduction

Schizophrenia is one of the most common global mental diseases, with prevalence of 1%. It is a major cause of disability and affects patients in the quality of life and work as well as interpersonal and self-care functioning. Moreover, the schizophrenic are under an increased threefold risk of premature death and shortened life expectancy of 10–20 years [1–3]. As the improvement psychosis treatments, there has been an increasing awareness of the need for high quality physical health care for the schizophrenic [4]. 

Compared to the increasingly significant recognition and management of obesity and metabolic problems, the appreciation of bone health has lagged behind. Osteoporosis is characterized by decreased bone stiffness, as signified by low bone mineral density (BMD), vertebral or nonvertebral fragility fractures, and disruption of bone microarchitecture. It is a significant health problem afflicting the global people [5, 6], and the female cases were more than male ones (5:2) older than 50 years of age, which brings about a disease burden of around *£*1.8 billion in the UK and *£*30 billion in whole Europe [7]. Although the high incidence rate of osteoporosis and osteoporotic fractures in the schizophrenic patients was first reported about 20 years ago [8–10], related reports about the increased risk of osteoporotic fracture and earlier onset of osteoporosis in the schizophrenic patients are seldom published [11]. Recently, many papers have presented convincing evidence that decrease of bone mineral density is related to schizophrenia particularly in patients treated with psychotropic medication [12–15]. 

In this paper, we will review the osteoporosis epidemiology and risk factors of schizophrenia to investigate whether antipsychotics can contribute to the development of osteoporosis. Our discussion focuses on the possible mechanisms involved and the clinical implications of such a relationship. And some prevention measures for osteoporosis in the schizophrenic will bring forth.

## 2. Epidemiology of Osteoporosis in Schizophrenic Patients

Comparing to normal people, patients with chronic schizophrenia actually show an exceedingly high prevalence of osteoporosis and bone fracture, and there have been a large amount of reports which indicate that bone mineral density decreased markedly in them [16, 17]. For instance, a study comparing ultrasound bone mass in 73 patients with schizophrenia on antipsychotic therapy with a matched number of healthy controls demonstrated increased bone loss in the former [18]. In addition, a UK General Practice Research Database study including 29,889 matched controls reported a statistically significant association between prolactin-raising antipsychotics and fractures; it showed that the relative risk of fracture at any site was increased 2.5-fold in premenopausal women with psychotic disorders, while hip fracture rates were increased 5.1-fold and 6.4-fold in older women and men, respectively, [19]. A Danish study also found a 1.2-fold increased fracture risk in those taking antipsychotics [20], while a Dutch population-based case-control study reported a 1.68-fold and 1.33-fold increased risk for hip or femur fracture for current and past users of antipsychotics, respectively [12]. Furthermore, a large case control analysis, including 22,250 hip/femur fractures with an equal number of controls, provided evidence that patients on antipsychotics were at an increased risk of hip/femur fractures, regardless of the antipsychotic drug prescribed [16].

## 3. Risk Factors for Osteoporosis in Schizophrenic Patients

Since such a high incidence of osteoporosis in schizophrenia, we should consider the possible risk factors involved and the clinical implications of such a relationship. The risk factors can be grouped into genetic and modifiable risk factors. 

### 3.1. Genetic Risk Factors

Genetic risk factors include female sex, old age, White or Asian race, or family history of osteoporosis [21]. In a study carried out in 2006 with schizophrenic patients treated with haloperidol, Jung et al. obtained results that female patients, instead of the male, showed significantly lower BMD, using densitometry techniques by DEXA (dual-energy X-ray absorptiometry), than the normal controls in all bone regions studied. Therefore, BMD loss in in schizophrenic patients tended to differ by gender [22]. But the result is in disagreement with several studies of psychiatric patients, which significantly found lower bone mineral density in men than in women associated with neuroleptic use [23, 24]. These gender differences may owe to the age differences in onset of schizophrenia [25]; that is to say, men have an age at onset approximately 5 years younger than that in women, and illness-related factors including medication will therefore have a longer-lasting impact on male patients. An alternative explanation suggested by Hummer and Huber is that women with schizophrenia take better care of themselves with regard to adequate nutrition and exercise than men and therefore have less osteoporosis [26]. Bone density in elderly persons is highly relevant to the risk of osteoporotic fracture was recognized by many years ago [27]. Ethnicity and family history of osteoporosis are important factors influencing the incidence of osteoporosis; also, Cauley reported rates of fragility fracture differ depending on race/ethnicity and are typically higher among those of White race [28]; Rybakowski et al. improved the functional polymorphism −1149 G/T (rs1341239) of the prolactin gene, and the G allele was associated with a diagnosis of schizophrenia in antipsychotic-induced osteoporosis [29].

### 3.2. Modifiable Risk Factors

Modifiable risk factors include low body mass index (<20–25 m/kg^2^), smoking, physical inactivity, poor dietary calcium intake, vitamin D deficiency, symptoms of the disease, and certain psychotropic medications [21, 30]. Liu et al. recently conducted a systematic review on osteoporosis risk factors in men and determined that the most important risk factors include age over 70 years old and low body mass which was in the general population [31]. The similar result was found by Hummer et al. in schizophrenia patients [23]. Smoking is particularly another important risk factor in schizophrenia, as much as 64% of patients with it have been known to smoke on a daily basis [32]. It was first recognized as a risk factor for osteoporosis in the mid-1970s [33], Law and Hackshaw conducted a meta-analysis and report a strong correlation between cigarette smoking and low BMD or hip fracture in postmenopausal women; they reported that one in eight fractures is accountable to smoking and that smoking increases lifetime risk of osteoporotic fractures from 12% to 19% in women up to the age of 85 years and from 22% to 37% to the age of 90 years [34]. The influence of physical inactivity on bone mineral density might be a consequence of the behavior of patients caused by negative, depressive. Patients with negative and depressive symptoms are likely to be physically inactive and show less tendency to go outside, which could in turn lead to lower 25-hydroxy-vitamin D3 levels while 25-hydroxy-vitamin D3 is a determinant of BMD in children and adolescents [35]. Positive symptoms such as paranoid delusions, on the other hand, can lead to an erratic intake of food, leading to nutritional deficits, as well as poor dietary calcium intake, vitamin D deficiency. Both types of behavior, therefore, could have a negative impact on bone mineral density.

## 4. Antipsychotic-Induced Osteoporosis and the Mechanism

**Figure 1:**
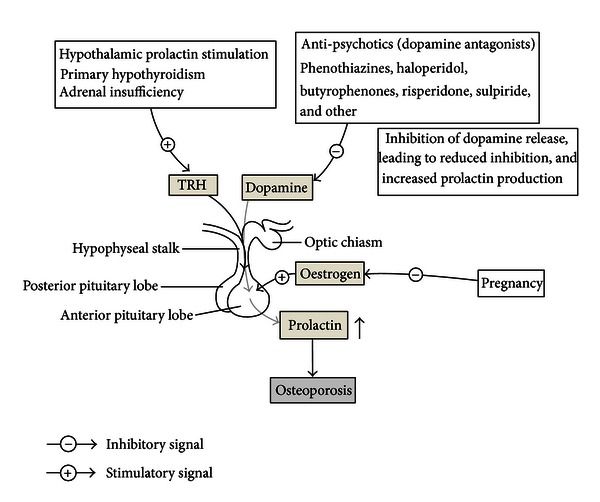
The physiological of antipsychotic-induced osteoporosis.

Except an increased prevalence of traditional risk factors, such as reduced physical activity, increased smoking, reduced calcium and vitamin D intake, as well as disease-specific factors, and certain psychotropic medications (Figure 1).

### 4.1. The Physiological of Antipsychotic-Induced Hyperprolactinemia

The mechanisms of antipsychotic-induced osteoporosis are complex, and the most possible one may be the hyperprolactinemia. Prolactin (PRL) is a 23 kDa polypeptide hormone secreted by the lactotroph cells of the anterior pituitary gland. Prolactin homeostasis is the result of a complex balance between positive and negative stimuli, deviating from both external and endogenous environments. Antipsychotics are the most common cause of pharmacologic hyperprolactinemia, and the majority of antipsychotic agents cause hyperprolactinemia [36]. There have been many studies that presumed a correlation between the decrease of bone mineral density found in patients with schizophrenia and hyperprolactinemia caused by long-term medication with antipsychotics also [26, 37, 38]. Cross-sectional studies have indicated that the prevalence of hyperprolactinaemia ranges from 42–93% in women and 18–72% in men [37]. Most of conventional antipsychotics can cause prolactin elevation, Marken reported treatment with conventional antipsychotics in patients with schizophrenia has been shown to increase serum prolactin concentrations 5–10 times above that of healthy control subjects in 1992 [39]. While among atypical antipsychotics, hyperprolactinaemia is well pronounced with by risperidone and paliperidone, followed by amisulpride [40, 41]. In the USA, Montgomery et al. report that hyperprolactinaemia occurred with all antipsychotics in their trial (risperidone 91%, olanzapine 40%, quetiapine 22%, and clozapine 11%) [42]. 

The mechanism of hyperprolactinemia is that antipsychotics block the dopamine D2 receptor of lactotrophs in the anterior pituitary and the prolactin secretion inhibiting function and consequently causes hyperprolactinemia [43]. Dopamine, secreted in hypothalamic periventricular zone (periventricular nucleus and arcuate nucleus) and released from neuronal projections in the median eminence, reaches the anterior pituitary gland through portal vessels (system known as “tuberoinfundibular dopamine pathway” or “TIDA”). The dopamine-mediated inhibition of prolactin secretion occurs through the binding of D2 receptors on the membrane of lactotroph cells and involves several signal transduction systems, resulting in inhibition of prolactin gene transcription and reduction of prolactin synthesis and release. 

It appears that risperidone and amisulpride are the main atypical antipsychotics associated with statistically significant increases in serum prolactin. As mentioned previously, amisulpride and risperidone penetrate the blood brain barrier poorly and, therefore, reach much higher concentrations in plasma than the CNS [44]. One study demonstrated that in amisulpride- and risperidone-treated rats, D2 receptor occupancy was higher in the pituitary (peripheral) than the striatum (central), with doses of amisulpride sufficient to induce 25% D2 receptor occupancy at the striatum inducing 100% D2 receptor occupancy at the pituitary gland. This may explain why amisulpride and risperidone seem to be associated with increased risk of hyperprolactinemia compared with other antipsychotic drugs such as clozapine, olanzapine, and quetiapine [45–47]. A positron emission tomography examination of D2 occupancy in the pituitary and temporal cortex supported this explanation and suggested that the greater rise in prolactin with risperidone may be due to the drug elimination from the brain by P-glycoprotein [48].

### 4.2. The Consequences of Hyperprolactinaemia

With the advent of prolactin sparing antipsychotics, ample consideration needs to be given to the physiological consequences of hyperprolactinaemia in schizophrenic patients. Hyperprolactinaemia has direct effects on the brain and on other organs. Direct consequences include galactorrhoea. Indirect consequences of hyperprolactinaemia include oligomenorrhoea or amenorrhoea, erratic or absent ovulation, sexual dysfunction, reduced bone mineral density, and cardiovascular disease [49].

Antipsychotics-induced hyperprolactinemia may influence bone metabolism in two ways. On one hand, hyperprolactinemia might directly affect bone turnover by stimulating bone resorption relative to bone formation [50, 51]. When recombinant prolactin was administered to pregnant rats, there was a 30% decrease of alkaline phosphatase in the newborn pups, despite normal parathyroid hormone and calcium concentrations. This appears to result from a direct suppressive effect of prolactin on rat osteoblast as demonstrated in primary cell cultures. Histology of the newborn pup bone showed reduced calvarial bone and reduced endochondral ossification. By contrast, the increase ratio of prolactin, which mirrored the increase of lactation in animal models, is associated with an increase of intestinal calcium absorption in animal models [52]. As a result, the calcium homeostasis may be improven. The importance of prolactin has been further illustrated in the prolactin receptor mouse knockout, which has marked hyperprolactinaemia and a decrease in bone formation rate and reduced bone mineral density, as measured by DXA [53]. The molecular mechanisms for these direct effects are not fully understood but may involve RANKL as it has been shown that prolactin can enhance production of mRNA for RANKL [54].

On the other hand, prolonged hyperprolactinemia may cause hypogonadotropic hypogonadism [55], resulting in suppression of gonadotropin-releasing hormone (GnRH) secretion in the hypothalamus and diminished secretion of luteinizing hormone (LH) and follicle-stimulating hormone (FSH) by the pituitary gland, resulting in a diminished secretion of sex hormones and ultimately in changes in bone metabolism [56]. Oestrogen is a well-known and prominent factor in bone metabolism. Hypoestrogenism leads to an increased risk for osteoporosis. Oestrogen inhibits osteoclastic activity while increasing gene expression in osteoblasts and increasing the level of type I collagen produced by osteoblast cells. Furthermore, oestrogen affects the synthesis of 25-OH-D and the absorption of calcium in the intestine. By contrast, there are fewer studies of testosterone in the context of bone metabolism. However, due to its effect on osteoblastic activity, low levels, of testosterone are correlated with osteopenia and/or osteoporosis. In the same way, dehydroepiandrosterone (DHEA) and its sulfate (DHEAS), important androgen as well as oestrogen precursor, are known to correlate with BMD [57].

## 5. Prevention and Treatment

Despite the established evidence of antipsychotic-induced hyperprolactinaemia, and hyperprolactinaemia associated with osteoporosis, published guidance on the monitoring and management of elevated prolactin levels in patients receiving antipsychotic treatment is lacking. The efficacy of preventive measures and treatment strategies to avoid or treat osteoporosis and reduce the risk in patients on antipsychotic medications could be the subject of health strategy studies.

### 5.1. Recommendations for the Prevention of Osteoporosis

It should be in line with established preventive measures, although there are many risk factors for osteoporosis—some of which can not be changed, including being female, being Caucasian or Asian, and having a direct relative who has had an osteoporotic fracture. However, there are many risk factors can be addressed, which can allow patients with schizophrenia to take control of their bone health and help prevent osteoporosis, including having a well-balanced diet, making lifestyle changes like stopping smoking, and minimising alcohol and caffeine intake. Vitamin D therapy is a recommended clinical practice in patients suffering from a decrease of bone mineral density [58, 59]. A prophylactic addition of vitamin D to the treatment of patients with schizophrenia who suffer from vitamin D deficiency would avoid loss of bone mineral density [60]. 

Clinicians should ask questions to detect risk factors before treatment start and then give patients relevant information. For prolactin-raising antipsychotics, it has been recommended that patients are questioned on possible prolactin-related effects until a stabilized dose is achieved [61]. In addition to the above trial data, Peveler et al. recommended that all patients prescribed antipsychotics should undergo prolactin screening at initiation of a three-month treatment [62]. In patients with elevated prolactin levels, other potential causes of hyperprolactinaemia should be ruled out [45, 61], several management options are available to counteract the effects of antipsychotic-induced hyperprolactinaemia [45], Such as dose reduction, switching drug or adding the partial agonist aripiprazole, that include the use of dopamine agonists. The addition of a D2 receptor agonist to an existing antipsychotic treatment is another management option. The dopamine agonist, bromocriptine, corrects elevated prolactin levels and has been shown to increase mean bone mineral density [63]. However, bromocriptine may be associated with adverse effects such as postural hypotension and gastrointestinal symptoms [45]. Adjunctive aripiprazole, which is a partial agonist, concomitant treatment may correct prolactin levels reduced prolactin [64]. Within 12 weeks, prolactin levels had fallen, and there was improvement in libido after switched or added aripiprazole to the medication of 27 patients. In males, both erectile and ejaculatory difficulties improved. In females, menstrual dysfunction was also significantly improved [65]. 

### 5.2. Recommendations for the Treatment of Osteoporosis

Once diagnosed, treatment of osteoporosis should be initiated in close cooperation with other multidisciplinary teams in order to reduce falls and prevent fractures. Except safer exercise options and falls prevention, there are a number of medications to treat osteoporosis and help reduce the risk of fractures. Drugs to treat osteoporosis can be grouped into two categories. The first ones are comprised of agents that limit the rate of bone loss. These drugs, also known as “anti-resorption drugs,” may decrease the rate at which osteoclasts reabsorb bone. The other category of drugs or the so-called “bone forming drugs” may promote bone formation. Recently, only antiresorbers are approved in the United States by the FDA for treating osteoporosis, and none of the drugs in this group has proven to be effctive yet.

Bisphosphonates are medications that slow the breakdown and removal of bone (i.e., resorption). This kind of drugs is widely used for the prevention and treatment of osteoporosis in postmenopausal women. It is also recommended for men or postmenopausal women with severe hip or spine osteoporosis. Zoledronic acid or raloxifene may be suggested for patients who cannot tolerate oral bisphosphonates, or who have difficulty in taking the medication, including an inability to sit upright for 30 to 60 minutes. Parathyroid hormone is another medication that can be used to treat osteoporosis. Oestrogen therapy for the development and maintenance of bone health has been well documented. The positive role of oestrogen replacement in prevention and treatment of osteoporosis among postmenopausal women is widely accepted, while oestrogen use among premenopausal females is not yet established as an effective treatment. Many researchers have identified increases in BMD and slightly increases the risk of breast cancer, stroke, and blood clots in premenopausal amenorrheic females treated with hormone replacement [66, 67]. Due to the incidence of breast cancer, strokes, blood clots, and heart attacks may increase in the women who take estrogen; the FDA recommends that women should take the lowest effective dose for the shortest period possible. Estrogen should be considered only in the situation that the patient is at a significant risk for osteoporosis, while patients who do not have any estrogen should be considered to take osteoporosis medications as the first choice. 

## 6. Conclusions

In conclusion, the combined use of typical antipsychotics including atypical risperidone and amisulpride increases the incidence of hyperprolactinemia in patients receiving treatment, which in turn causes a reduction in BMD but an increased risk of fracture. Evidence suggests that this mechanism of action results through a “direct pathway” in osteoblast cells independently and more prominently through an “indirect pathway” via hypothalamic-pituitary-gonadal axis [68]. Prolactin screening programs in patients receiving long-term treatment (>6 months) with these types of antipsychotics may be necessary, even in the absence of clinical symptoms relating to hyperprolactinemia, in order to identify those with the highest risk of developing medication-induced osteopenia and osteoporosis. Further controlled studies and adequate guidance are essential to increase awareness and understanding of the impact of antipsychotic-induced hyperprolactinaemia on physical health in schizophrenia.
